# Physiotherapy for vegetative and minimally conscious state patients: family perceptions and experiences

**DOI:** 10.3109/09638288.2015.1005759

**Published:** 2015-09-04

**Authors:** Julie Latchem, Jenny Kitzinger, Celia Kitzinger

**Affiliations:** ^a^ School of Social Sciences, Cardiff University, Cardiff, UK; ^b^ School of Journalism, Media and Cultural Studies, Cardiff University, Cardiff, UK; ^c^ Department of Sociology, University of York, York, UK

**Keywords:** Disorders of consciousness, family, physiotherapy, vegetative

## Abstract

*Purpose*: To examine family perceptions of physiotherapy provided to relatives in vegetative or minimally conscious states. *Method*: Secondary thematic analysis of 65 in-depth narrative interviews with family members of people in vegetative or minimally conscious states. *Results*: Families place great significance on physiotherapy in relation to six dimensions: “Caring for the person”, “Maximising comfort”, “Helping maintain health/life”, “Facilitating progress”, “Identifying or stimulating consciousness” and “Indicating potential for meaningful recovery”. They can have high expectations of what physiotherapy may deliver but also, at times, express concerns about physiotherapy’s potential to cause pain or distress, or even constitute a form of torture if they believe there is no hope for “meaningful” recovery. *Conclusion*: Physiotherapists can make an important contribution to supporting this patient group and their families but it is vital to recognise that family understandings of physiotherapy may differ significantly from those of physiotherapists. Both the delivery and the withdrawal of physiotherapy is highly symbolic and can convey (inadvertent) messages to people about their relative’s current and future state. A genuine two-way dialogue between practitioners and families about the aims of physiotherapeutic interventions, potential outcomes and patients’ best interests is critical to providing a good service and establishing positive relationships and appropriate treatment.

**Implications for Rehabilitation**
- Families of people in PVS or MCS consider physiotherapy as a vital part of good care. Clear communication is critical if therapeutic input is withdrawn or reduced.
- The purpose of physiotherapy interventions can be misinterpreted by family members. Physiotherapists need to clarify what physiotherapy can, and cannot, achieve.
- Families can find some interventions distressing to witness – explaining to families what interventions involve, what they can expect to see (and hear) may be helpful.
- Physiotherapists and families can attribute different meanings to physiotherapy. Physiotherapists need to identify how families view interventions and modify their explanations accordingly to enhance information sharing.

## Introduction

Brain injury is the leading cause of death and disability in young adults in the western world. Mortality ranges from 30% to 50% in those sustaining severe injuries, and approximately 30% of those who survive are left with significant and long-term neurological impairments [1] which includes, at the most extreme end, a disorder of consciousness [2]. “Disorder of consciousness” is an umbrella term referring to coma, the vegetative and the minimally conscious states (VS and MCS) – where the patient has no, or only minimal and intermittent, awareness of self and environment [3,4]. Such a disorder can be a temporary or long-term condition: some patients will move through stages of coma, vegetative and minimally conscious states and emerge into full awareness while others will remain in a vegetative or minimally conscious state for the rest of their lives [5].

The role of physiotherapists in acute, rehabilitative or long-term care and treatment of these patients is recognised and discussed in the general medical and therapeutic literature [6–8] and specified in guidelines such as those produced by the Royal College of Physicians [5] in the UK, the National Health and Medical Research Council [9] in Australia and the Multi-Society Task Force [10] in the USA. These guidelines identify physiotherapy as a key discipline within the multidisciplinary team assessing, diagnosing and managing this patient group and recommend the delivery of physiotherapeutic interventions such as manual secretion clearance techniques and suctioning (with the aim of reducing the risk of respiratory infections or to treat those which have already occurred) and casting, splinting, stretching, positioning and standing interventions (with the aim of managing abnormal tone, maintaining joint range of movement and muscle length and preventing contractures) [5,9,10].

Physiotherapy treatments for vegetative and minimally conscious state patients may also aim to facilitate arousal and postural control [8,11], increase pulmonary ventilation and improve circulation [12]. Physiotherapists may also be involved in the delivery of multisensory stimulation programmes aiming to increase level of arousal and awareness through the stimulation of the reticular activating system [13–15] and serve as part of a multi-disciplinary team evaluating the consciousness of patients [5].

However, the clinical efficacy of these treatments for this patient group is considered contentious due to a current lack of evidence [8,15–18]. Research investigating the efficacy of either specific interventions such as sensory stimulation programmes [15] or multiple physiotherapeutic treatments [8] come to this same conclusion. However, it is also noted by several authors that although there is a lack of evidence which supports these interventions, neither is there evidence which proves them to be ineffective [8,15]. In addition, physiotherapy is not alone in lacking evidence of its effectiveness: there are also gaps in the assessments of many of the interventions performed by other health professional groups for this (minority) patient group [19,20].

While one way to talk about the role of any treatment is to study its effectiveness in achieving its goals (or review the evidence about this), another way is to look at how the treatment is experienced and understood by the patient or their carers/families. Both types of research are crucial to good service delivery – and it is to this second type of literature that this article contributes.

There is an internationally established literature base within health, social care and the social sciences which explores “lay” perspectives of health and illness and examines the experiences of both patients and carers [21–26]. This has informed a small, but emerging literature on patient and family understanding and experiences of physiotherapy, which recognises the insights that can arise from attending to lay accounts; illuminating the interconnectedness of lay people’s perceptions of treatment and interactions with physiotherapists, clinical outcomes and patient/family satisfaction [27–33].

This article adds to this emerging literature through its focus on “lay” perspectives on physiotherapy treatment of vegetative and minimally conscious state patients. It is not possible to access patients’ perspectives in such cases – but it is possible to investigate their *families*’ point of view. Understanding family perspective is important because research highlights that families face profound challenges in dealing with, and making sense of what is happening to their relative [34–39]. Family members are a key support for the patient and a critical source of information. Good communication with families is an important part of establishing positive care relationships and delivering appropriate care treatment for these patients. This article therefore explores what families have to say about the physiotherapy given to (or withdrawn from) their relative, what meanings they attribute to these therapeutic practices and reflects on the implications this has for day-to-day family–therapist interaction and service provision.

## Methods

The research reported here is based on secondary analysis of a major in-depth interview study of the experiences of families with a severely brain-injured relative. The original study focused on family experiences of decision-making about serious medical treatments – but interviews were wide-ranging and proved to be a rich source of data about interviewees’ perceptions of many different issues, including physiotherapy. The significance of the physiotherapy data was identified by the first author [J.L.] (herself a physiotherapist) when she was analysing the interview transcripts to answer another research question [40]. Following discussion with the second and third authors [J.K., C.K.] (who conducted the original interviews), it was decided to conduct a secondary analysis to focus on experiences and perceptions of physiotherapy.

Secondary data analysis is now a frequently used methodology in health research as it maximises the use of existing data and minimizes intrusion and “research fatigue” for research participants [41,42]. In this case working in collaboration with the researchers who collected the original data ensured that the knowledge of these researchers could be combined with the interests and expertise of the new researcher as the data was mined to address a new question.

### Data collection

The original study was conducted between 2010 and 2014 and involved interviews with 65 family members who have (or had) a relative in a vegetative or minimally conscious state. Research participants were mostly interviewed one-to-one, but occasionally in pairs (e.g. a husband and wife asked to be interviewed together, as did a mother and daughter). The majority of interviews lasted between 2 and 4 h (with breaks). Most people were interviewed just once, but some were interviewed twice (or even three times) – either weeks apart (if they simply wanted to talk longer) or, in some cases, years later, when significant changes had occurred and interviewees wanted to talk about new aspects of their experience or perspective. Interviews were transcribed orthographically and the data were analysed thematically to identify recurrent patterns (themes). Existing publications based on that original thematic coding have examined issues such as experience of decision-making [35], perceptions of life and death [37], attitudes to treatment withdrawal [38], family stresses [39], views of the law [43] and understandings of diagnosis [44]. For the purposes of this article, we focus on those parts of the interviews where participants referred to physiotherapy.

### Sampling and recruitment

Recruitment of participants was the responsibility of the second and third authors and was initially carried out via their own social contacts and advertising via brain-injury support groups. This was made possible in part as these academics (who are sisters) are not only experienced social science/health researchers but they also have a close relative who is severely brain injured. After the initial few interviews, the researchers also used snowball sampling and contacted people via care homes and various professionals and got in touch with some who had spoken publicly about their experience.

Of the 65 family members interviewed, 41 were women and 24 were men. Interviewees were the patient’s mother or father (*n* = 18), their sibling (*n* = 15), spouse/partner (*n* = 11), adult son or daughter (*n* = 11) or other family members involved (e.g. sister-in-law or daughter-in-law; *n* = 10). Interviewees were mainly early to late middle age and the patient they were talking about had mostly been injured in their teens, early adulthood or middle age (only a quarter of the patients were over 50 at the time they were first injured). The relatively young age of the patient, and their family members, is a distinctive feature of many serious brain injuries and it tends to be younger patients who may be sustained long term in the vegetative or minimally conscious state. All except two of the patients were being treated in the UK (although some had initially been injured and received emergency treatment abroad e.g. while on a ski-ing holiday). Most patients were currently either VS or MCS (some had died by the time of interview and others had emerged with severe neurological deficits).

### Data analysis

Data relevant to physiotherapy was identified both via reading the full transcript of interviews and by double-checking all sections identified using key search terms. The obvious search term to use was “Physio*” – where the “*” indicates a search term which will capture multiple versions of a term, e.g. searching “physio*” captures “physio”, “physiotherapy” and “physiotherapist[s]”. In addition, the search was supplemented with the search terms: Stretch*, Splint*, Spasticity, Thera*, Tilt table, Exercise*, Rehab*, Trach*, Occupation*, Secretion*, Diet*, Breath*, Swallow*, Infection, Pneumonia, Suction, Chest, Speech Language and SMART.

All data extracts relating to physiotherapy were then examined in detail. In analysing the resulting data, we took a “realist” approach to the data and analysis, seeking to report the reality of participants’ experiences and the meanings attributed to them as expressed in interview [45] and used thematic analysis to identify recurrent patterns (themes) following the procedure described in Braun and Clarke [45]. Thematic analysis is appropriate for use in research which seeks to identify the range of perceptions, experiences and their meaning [46] – as is the aim here.

Data coding and initial themes related to physiotherapy were initially compiled by the first author [J.L.]. The interview excerpts were read and re-read in order to become familiar with the data and context. Notes were made and initial categories highlighted. An index of the initial categories was then produced and the data re-read with these in mind and systematically searched for reoccurrence of the identified categories. Particular attention was paid to any experiences or perceptions that were contradictory or unusual (deviant case analysis). Sections of data at this stage were coded, either line by line or in chunks and each piece of data was then revisited, assigned and assembled into a theme. New themes emerged during this process as data that did not fit previously identified themes were re-considered and subsequently themes were constructed, deconstructed and reconstructed. The data analysis was initially carried out by [J.L.] and final themes developed following discussion with [J.K. and C.K.].

### Ethical considerations

Research ethics committees at the Universities of [Cardiff and York] initially approved the study which subsequently gained NHS approval from Berkshire Research Ethics Committee (09/H0505/66). Participants in the primary study gave informed consent for their anonymised data to be shared with members of the Cardiff-York Chronic Disorders of Consciousness research group. Ethical approval for the secondary analysis was gained from Cardiff University.

Pseudonyms are used throughout and care has been taken to remove unique identifying details. However, due to the relatively small numbers of people with a relative in this situation, there is still a risk that some participants could be identifiable and therefore some quotes are left unattributed and at times, their gender and/or roles may have been changed to increase anonymity and prevent jigsaw identification (refer [47,48] for further details regarding our anonymising strategy).

## Results

Physiotherapy formed a key part of family members’ accounts about the care and treatment of their relative. It was often the focus of spontaneous, detailed and passionate comment and intimately intertwined with interviewees’ assessment of the quality of their relatives’ care and their hopes and fears for the future. The significance accorded to physiotherapy is striking given that this was not the focus of the original study and interviewees were not usually directly asked about it.

Analysis of the nature of interviewees’ comments about what they thought physiotherapy provided identified six key themes. These were: “Caring for the person”, “Maximising comfort”, “Helping to maintain health/sustain life”, “Facilitating progress”, “Identifying or stimulating consciousness” and “Indicating potential for recovery”. These six themes are unpacked in the first part of findings section below. We then explore two additional themes: how interviewees felt about the *withdrawal* of physiotherapy services and their reflections on the potential of physiotherapy to cause pain and distress, which could even be considered to constitute a form of “torture” once relatives felt there was no longer any realistic hope of recovery that the patient would consider “meaningful” or “worthwhile”.

### What physiotherapy can do: family perceptions

#### Caring for the person

Physiotherapists were often singled out for their ability to offer individualised, person-centred care in a context where the vegetative or minimally conscious state patient risks being treated as “just a body”. They were often considered to be attentive to the individual and interested in knowing about the person, prior to injury. The way in which physiotherapists interacted with patients was sometimes experienced by families as helping to re-personalise their relative. Lily, who felt abandoned on a hospital side ward with her brother in a minimally conscious state soon after his accident, described what a difference had been made by an attentive physiotherapist:

[we had] all these pictures […] of [my brother] on nights out and doing things, so people could remember that he wasn’t just this horribly smelly vegetable thing […] And she [the physio] was the only one that started looking at these pictures and she was interested in them, you know, and asking about them and talked to [my brother]. But no one else did. They came in and they went out. And it was so lonely*.* (Lily, sister)

However, conversely, when physiotherapists failed to show an interest in patients and talked about and over them, this was perceived as both disrespectful and depersonalising.

#### Maximising comfort

Interviewees spoke at length about the importance of physiotherapeutic interventions in promoting patients’ comfort. Passive range of movement and stretches were identified as maintaining joint range and preventing contractures. Despite a lack of research evidence regarding the efficacy of such physiotherapeutic treatments [8,16–18], family members experience such interventions as valuable such that delaying, withdrawing or withholding physiotherapy was often seen as compromising the patient’s well-being. One mother, for example, talked about a time when her son (who had been in a vegetative state for several years) was denied physio and said: “we just felt this was time wasted and he had gone backwards”. Daisy, the sister of a minimally conscious man described how:

We knew he needed to have his arm physio-ed however many times a day, and they left him for ten days […] [We said] “You have to continue doing this because otherwise he will deteriorate” […] And of course then by the time they started doing it he'd got much tighter and he'd started having contractures. (Daisy, sister)

Physiotherapists were also appreciated for the attention they gave to positioning. Rose, for example, spoke about how physiotherapists had taken responsibility for making sure that her relative was comfortable at a time where she felt he was being dismissed by other staff as having no feeling at all. She said:

They couldn’t be bothered to put him in his chair properly. And he, clearly, was looking distressed. […] And in the end, I did my nut […] Not one time did any of the ordinary staff that should have been dealing with it come to me and say, “We are so sorry”, […] It was the physio girls who came and said, “I promise you, Rose, I will come in and check that Sid is comfortable”. (Rose, relative)

Families often found the look and sound of a relative struggling to breathe profoundly distressing and were also grateful for interventions such as suctioning when the patient had pneumonia for example (as is common in this patient group).

She was very, very, obviously distressed by the fact that she literally couldn’t breathe […] so I asked that they would kind of suction her chest. And they did do that quite swiftly and she was a bit more comfortable after that. (Sonia, daughter)

#### Help to maintain health/save life

Over and above this, physiotherapists were seen to have a crucial role in maintaining life itself by helping to prevent and treat chest infections. Family members referred to suction being provided by a variety of staff – physiotherapists, nursing and care workers. However, several spoke about the difference between the chest care provided by physiotherapists and other health care staff, commenting that chest care (suction and manual chest clearance techniques) provided by physiotherapists seemed to be more “effective” than that provided by others.

#### Facilitate progress

Physiotherapists were seen not only as reducing discomfort or complications/threats to life but as a crucial part of the team assisting the patient (potentially) to achieve future functional recovery. Maintenance of joint range of movement and muscle length was viewed as critical by some interviewees who hoped that feet that were not allowed to turn inwards might one day be used for walking, or that a hand that could grasp might one day be able to press a call button. Several interviewees had extremely optimistic imaginings of what might be possible and focussed on physiotherapeutic interventions as a route through which some functional recovery would be achieved. One father, for example, persisted in believing that his daughter (diagnosed as being in a permanent vegetative state) would eventually “wake up”. Although well aware that he might be criticised for this by clinicians and other family members, he refused to accept that his daughter had severe brain damage, focussing instead on the obvious, visible problem of his daughter’s spasticity and how physiotherapy might be able to resolve this:

I don’t want this to get back to the rest of the family, alright? […] [but] I think that Jane will mentally be okay, talk and the rest of it. But the problem is Jane’s legs are like that all the time [miming turning feet inwards]. They keep trying to straighten them out. (John, Father)

#### Identify or stimulate consciousness

The potential of physiotherapy to help recovery was also explicitly linked to family observations that physiotherapy aided in the detection of, or actually enhanced, apparent displays of awareness/consciousness, as Rose observed: “when Sid was put on the tilt table he woke up […] so we all wanted Sid on it more”. Several interviewees believed that physiotherapists were particularly good at noticing consciousness or that their treatments *enabled* awareness to be more readily detected because positioning could aid alertness or because pain and exhaustion could mask consciousness or limit a patient’s ability to respond.

In the acute ward at [London Hospital] they were very good in terms of physio […] and as a result he was much more flexible and comfortable. And I think that’s why he seemed more aware. (Daisy, sister)

#### Indicate potential for meaningful recovery

The meaning of physiotherapy – and what it might or might not detect or deliver – was a key element in interviewees’ accounts. Some talked about having their expectations raised through responses witnessed during physio (such as eye opening) or through the optimism of a physiotherapist. As one mother, talking about a physiotherapist treating her son in PVS, remarked: “she said she was really excited about working with him, and gave the impression anything was possible”. In retrospect, some family members felt they had been misled as the hoped-for recovery had either not materialised or some consciousness had returned, but this had made the situation worse (e.g. the patient now seemed distressed). Balancing realistic prognostic expectations and hope for recovery in interactions with families is complex as some interviewees could also experience the delivery of a poor prognosis as insensitive. Felicity, for example, describes her outrage against a physiotherapist who, she felt, was prematurely dismissive:

He was just shockingly horrible […] the things he was saying about Nin. “Well, I can see it’s quite evident Nin hasn’t got any reactions, and I don’t think there’s much hope for an outcome”. In front of Nin, that Nin’s not going to get better! (Felicity, partner)

In contrast, Miggy (the mother of a boy in a permanent vegetative state) reports with gratitude her memory of a trusted physiotherapist who said simply “We’re not winning” – a remark rejected at the time, but later valued as helping the family finally to come to terms with the fact that the young man in question was never going to recover consciousness.

## When physiotherapy is delayed, withdrawn or withheld

Given the value placed by families on physiotherapy it is not surprising that provision was often a point of contention. Interpersonal conflict on a day-to-day level was often evident in the accounts (e.g. “I’ve had issues – mainly with the physio – and the lack of treatment and the way that treatment’s been applied”) and family members blamed gaps in physiotherapy provision for obstructing good treatment and accurate diagnosis. Patients in prolonged vegetative states were also seen as being at “the bottom of the heap” in the competition for resources. For example, Rhiannon, whose daughter had been vegetative for many years and is cared for at home, commented:

They [physiotherapists] would prefer to work with the people that are able because that reflects better on them when they get better, and they know that […] in cases like Amy, they're not going to get better. (Rhiannon, mother)

Her own view was that her daughter needed physiotherapy *more* than other people precisely because she was so entirely helpless, but Rhiannon believed the physiotherapy profession worked to other criteria:

As far as I’m concerned, Amy needs to be moved about and given physio because she can’t do it for herself. But, you see, the profession is making these decisions. (Rhiannon, mother)

For other interviewees, decisions to withdraw services were sometimes seen as premature (“writing off” the patient) or, even if accepted as appropriate, were profoundly significant because withdrawing physiotherapy signalled to family members that no further improvement was expected by clinicians. Withdrawing therapy was therefore experienced as “withdrawing hope”.

From that point onwards it’s fairly clear that […] there’s going to be no improvement […] you suddenly realise that they clearly think that this is pointless […] it feels like a kind of relegation. (Sonia, daughter)

Even when they recognised their relative as being in a *permanent* vegetative state, with no hope of recovering consciousness, the continuation of physiotherapy was also important to some relatives, as part of the overall provision of care. Cathy, whose brother was in PVS explains:

Although I didn’t think that [brother] had any awareness […] it was just torturous to think of this body being like left in his own mess, or allowed to become more spastic […] so he carried on having therapy all the time because although we thought his life should come to an end and although we thought he had no awareness or meaningful life, none of us could bring ourselves to still, not to extend full kind of compassion and care. (Cathy, sister)

The withdrawal of therapies could therefore be considered by family members to be a removal of, or reduction in, care provision. Another interviewee, Sonia, also felt that withdrawal of therapies was associated with a general downgrading of her mother’s care in the hospital setting:

The level of care for those patients who are no longer having therapeutic intervention was quite poor in terms of, you know, you’d turn up and find that mum’s […] hair was dirty and she just smelt […] she had dribbled and people hadn’t cleaned up her face and this kind of thing. […] It [withdrawal of therapy] certainly felt like that implied a downgrading of care for the remainder of the person. (Sonia, daughter)

## When physiotherapy becomes “pointless”: causing pain and distress and physiotherapy as “torture”

Although physiotherapeutic interventions were usually highly valued, they could be a source of ambivalence – and were sometimes rejected outright. Suctioning, splinting and use of the tilt-table could be perceived not *only* as contributing to comfort or as being “good” for patients but instead (or also) as painful, intrusive and distressing. Family members talked of patients “forced” to wear splints, and described the discomfort and restriction it placed on the already profoundly disabled individual, the “mummified” appearance of the splinted limbs, and the marks left on arms and legs. One interviewee described the tilt table as being “terrifying” for her daughter, another came to see its use as a source of “agony”, as “cruel” and “wrong”, a third acknowledged that the table was “meant to be positive” but commented “but it just looked so awful, like some horrible medieval torture implement and it was just so dreadful” (Cathy, sister). For Cathy, seeing her brother upright on the tilt table appeared “unnatural” and magnified her brother’s disabilities and physical disfiguration. She also talked about how distressing she found the eye opening stimulated through its use, because his open eyes were blank and “unseeing”.

Interviewer: Why is the image of him on the tilt table so much worse than him in the bed?

Cathy: I think because there was something in his face, not quite expression but, his eyes were open, […] it’s difficult to explain, it’s the eyes open that is actually much more distressing really, unseeing eyes. I find that really, I found that really difficult. (Cathy, sister)

Many interviewees also described their distress at witnessing suctioning. One, for example, talked vividly about the sounds of deep suctioning and the apparent distress of her relative: “the look of panic and horror when that whizzing sound – but then the panic and horror of choking”; another described her sense of helplessness in being unable to reassure her son in PVS during such procedures:

I’m sure I don’t need to tell you how distressing it is to watch a relative having their lungs suctioned, […] and there’s not a damn thing you can do to protect them or even explain to them or reassure them. You know, if it was a dog, you could stroke its ears and make soft noises at it. If it’s child you can cuddle it and say it’s all going to be better very soon, be brave. But for someone in a PVS state, there’s absolutely nothing you can do. (Josie, mother)

Others talk about suctioning with disgust and repulsion and hold long lasting vivid visual memories of the intervention. Cathy, recalling her brother in PVS receiving suction recalls:

It was revolting. They were just suctioning all this stuff off his lungs all the time. And I kind of can’t – I know this doesn’t make sense – so in my head I remember it as some kind of like horrible laboratory with all these glass jars full of all this green, brown, bloody stuff. Which can’t be quite – there can’t have been like lots of jars. But they were just like suctioning, continually suctioning all this stuff off his lungs. It was really – it just made me feel sick the whole time, I spent the whole time thinking I might throw up. (Cathy, sister)

For some interviewees invasive physiotherapy, however distressing it might be, was considered necessary and “cruel to be kind”, but for others (sometimes the same people interviewed a year or so later) such treatment had become an unjustified imposition and any gains that might be made were seen as ridiculously small. Imogen, for example, angrily rejected the suggestion from a health care professional (not a physiotherapist) assessing her husband that he might “benefit” from more physiotherapy.

She said to me, “Do you know, maybe he needs more physiotherapy and he needs this and he needs that and then…”, and then I said, “And then what?” And she said, “Then he’d be able to do…”, I said, “Yeah, what would he be able to do?” (Imogen, wife)

And, in the case of Cathy, observing her brother being suctioned, approximately four years after his initial brain injury, provided a significant trigger for her to question his quality of life and the purpose of continuing life prolonging treatment.

I think it was probably then for the first time that it even occurred to me that maybe it might be better if – or even, would it be such a terrible thing if – he did actually die? I think it was the first time I’d had that thought. But we were still trying for him not to. Putting all these sort of heroic efforts into keeping him alive. (Cathy, sister)

Given the poor quality of life anticipated by some family members physio was seen as pointless, or worse than pointless. In particular, the role of chest physiotherapy or suction in combating chest infections and prolonging physical existence could be viewed with intense ambivalence. On the one hand, chest physiotherapy was seen to reduce suffering, but on the other hand, it could be seen to extend it. Families often found it intolerable to hear a relative gasping for breath and wanted interventions even as they also wanted their relative to be “at peace”. One woman, for example, performed manual secretion clearance techniques and oral suction on her sister herself, even though a palliative pathway had been agreed. Another believed her relative would rather die of pneumonia than continue to exist in PVS but still insisted on suctioning because: “they’ve got to be treated because you cannot listen to that [the sound of them gasping and wheezing]. You can’t have them suffer”. Others, however, especially if they were aware of other ways of treating chest infections on a palliative pathway saw intrusions such as suctioning in these circumstances as a form of torture – “why can’t they just make him comfortable, and leave him be?”. Once alternatives were offered, such as palliative nursing for pneumonia, this could be welcomed with relief if clear best interests decisions had been made, and good support put in place. One woman, for example, when first interviewed was outraged by the poor prognosis communicated by clinicians (including a physiotherapist) and by suggestions that life-sustaining interventions might be inappropriate. However, when interviewed again, two years later, she had come to see continuing to sustain his life as “cruel” and some physiotherapy interventions as “pointless”.

Why give him physiotherapy when it hurts him? […] He’s on a palliative care pathway now and that is right, it’s about comfort and care. (Fern, partner)

## Discussion

This research highlights the range of roles attributed to physiotherapists by families, the significance of physiotherapy to their assessment of the overall quality of their relative’s care, and the intense feelings connected to the provision of such services. Family members look partly to physiotherapy for answers to questions about their relative such as: Are they conscious? Will they be helped to recover consciousness or a quality of life they would find worthwhile? Are they comfortable or in pain? Are they being neglected or caused unnecessary suffering? Are they being treated as “a person” and are they receiving appropriate treatment – by which they usually mean treatment that their relative would have wanted for themselves.

How families react to physiotherapy provision (or its lack) is underpinned by the importance of such concerns. It is informed by what family members have understood about physiotherapy based on what they have been told (or researched) about its role, their own beliefs about the patient’s level of consciousness and potential recovery, their views of what the patient would have wanted, the patient’s current pathway (e.g. whether the patient is on a palliative pathway) and what they witness during therapy itself (e.g. eye-opening or the visceral experience of seeing and hearing treatments such as suctioning).

Our research highlights the value and importance families place on how physiotherapists relate to patients and the messages this conveys about care and respect for “the person”, not just “the body”. Physiotherapists can play a very positive role in giving comfort to families in a situation that can be lonely and alienating, and in which families can feel that there is a lack of person-centred care.

The research also highlights the ways in which family members identify the specialist skills that physiotherapists bring, especially in relation to dimensions such as respiratory care and physical management, as well as linking physiotherapy interventions and physiotherapists to stimulating moments of awareness and enabling its detection – an effect and aim of physiotherapeutic treatment only scantily explored in the literature to date.

Communication and decision-making were key themes to emerge from our analysis. The research highlighted the importance of family input into decisions about physiotherapy treatment where family members often provide the only continuity of care as patients move between services over time. They also often spend many hours at the bedside, and can be an important source of information about the patient which could be used to inform treatment (e.g. in relation to tone and responses to the suspension of physiotherapy).

Above and beyond this it is important to consider the implications of our research for communication with families about physiotherapy interventions and services. Our analysis of families’ accounts revealed the significance of the messages conveyed by physiotherapy about potential recovery – of both cognitive and physical function. Physiotherapists need to be aware therefore that the way family members interpret both the purpose and potential effectiveness of treatments can vary significantly from their own. Physiotherapists (alongside other practitioners, commissioners and managers of service provision) have a particularly important role in discussing with families the implications of current clinical evidence and the meaning of eye-opening and other “responses” and discussing what physiotherapy probably can, and cannot, achieve. We hope that by identifying the range of ways in which families understand the physiotherapy given to their relatives this article will help practitioners modify their communication and enhance information sharing, and dialogue, with family members.

Our research also identified the issues that might need to be unpacked in discussion between service providers and families when consideration is being given to withholding or withdrawing physiotherapy services given both the practical, and the symbolic, implications of any such decision. Withdrawal of therapies is often accompanied by a change in the type of care provided and, often, in who provides it. The quality of basic care however should be consistent irrespective of whether the patient requires acute, rehabilitation, long-term or palliative care. As patients move through the patient pathway and as certain types of treatments are withdrawn, continuity of basic care quality and person centred care must be upheld so that families don’t feel that the care of their relatives is being downgraded or their relatives relegated to “the bottom of the pile”. Physiotherapists have an important role both in communicating with families during the process of withdrawing or reducing therapeutic input and in contributing to maintaining good basic care alongside the wider multidisciplinary team.

The above considerations and issues in relation to communication however need to be positioned in a broader ethical framework. Family members raise important ethical questions both about “the right to die” and about healthcare provision for patients in vegetative or minimally conscious states. On the one hand a concern with distributive “justice” might suggest that resources should be distributed to those who can most benefit – and physiotherapy provided to this patient group could be seen as treatment denied to others (such as those with less profound brain injuries). However, considering that survival in VS and MCS is fundamentally an iatrogenic condition, does the health service have particular responsibilities in relation to the support of such patients and their families? This is a question we pose for future debate. Policy makers and funders however need to engage with these issues now and consider the ethics of allocation of services to the most disabled, who might benefit only minimally, but who have so little capacity that a ‘small’ benefit might be proportionately massive. They also need to make visible who does make decisions about who to treat, and who not to treat and on what bases such decisions are being made.

Finally, our analysis highlighted the importance of discussion between families and professionals addressing the invasiveness of, and justification for, some physiotherapy treatments. Practices which may have become routine for professionals may look intrusive and cruel to family members and can leave them feeling distressed and helpless in their inability to offer explanation or comfort to their brain injured relative. While some treatments are delivered to prevent further complications or provide comfort and family members often recognise this, explaining to families what interventions involve and what they can expect to see (and hear) may be helpful. Alongside this, it is also important to acknowledge that while, the clinical diagnosis and medical understanding of PVS attests that these individuals are not aware of themselves and environment, it is possible that patients can be misdiagnosed – for example, some people diagnosed as vegetative may in fact be minimally conscious or can emerge from a vegetative state into a minimally conscious one. Some studies estimate that misdiagnosis is as high as 40% [49]. There is also some recent research which suggests that people in PVS are able to experience pain [50]. Such findings may add to family’s anxiety around invasive interventions or those which they perceive could be painful for their relative.

Finally, it is important to recognise that treatment is not lawful unless it is in the patient’s best interests – and that best interests includes consideration of what the person would have wanted. Families are crucial in any best interests decision-making as a source of information about the patient’s prior expressed wishes (e.g. in relation to life-sustaining interventions such as chest physiotherapy). Unwanted intrusive treatment can indeed be “torturous” (refer [51] and [52] for further work on families' perspectives on medical treatments). Physiotherapists need to be aware of whether or not these patients are on a palliative pathway and if so, whether their treatments are in keeping with this.

### Limitations

This study used secondary data collected for purposes other than the research question posed here. This has advantages, e.g. discussion of physiotherapy were spontaneous and the importance given to it by some interviewees entirely unprompted. However, further research with primary data collection focussed on physiotherapy would be able to further unpack some of the issues raised in this analysis. Interviews with physiotherapists, and observational research would add further value.

## Conclusion

Families consider physiotherapy and physiotherapists to play a critical role in the care of their relative. Their perspectives are dependent upon and inextricably linked to the level and extent of their relative’s consciousness in the present and the likelihood of (and beliefs about) “meaningful” recovery in the future. Understanding the variety of interpretations, families give to physiotherapy and its practices is critical to developing positive relations with families of people in vegetative or minimally conscious states and providing care to their relatives. Physiotherapists should be aware that both their action but also inaction – for example, the withdrawal of services – are symbolically powerful.
